# Pharmaceutical Pollution from Human Use and the Polluter Pays Principle

**DOI:** 10.1093/phe/phad012

**Published:** 2023-07-12

**Authors:** Erik Malmqvist, Davide Fumagalli, Christian Munthe, D G Joakim Larsson

**Affiliations:** Department of Philosophy, Linguistics & Theory of Science and Centre for Antibiotic Resistance Research in Gothenburg (CARe), University of Gothenburg, Gothenburg, Sweden; Department of Philosophy, Linguistics & Theory of Science and Centre for Antibiotic Resistance Research in Gothenburg (CARe), University of Gothenburg, Gothenburg, Sweden; Department of Philosophy, Linguistics & Theory of Science and Centre for Antibiotic Resistance Research in Gothenburg (CARe), University of Gothenburg, Gothenburg, Sweden; Department of Infectious Diseases, Institute of Biomedicine and Centre for Antibiotic Resistance Research in Gothenburg (CARe), University of Gothenburg, Gothenburg, Sweden

## Abstract

Human consumption of pharmaceuticals often leads to environmental release of residues via urine and faeces, creating environmental and public health risks. Policy responses must consider the normative question how responsibilities for managing such risks, and costs and burdens associated with that management, should be distributed between actors. Recently, the Polluter Pays Principle (PPP) has been advanced as rationale for such distribution. While recognizing some advantages of PPP, we highlight important ethical and practical limitations with applying it in this context: PPP gives ambiguous and arbitrary guidance due to difficulties in identifying the salient polluter. Moreover, when PPP does identify responsible actors, these may be unable to avoid or mitigate their contribution to the pollution, only able to avoid/mitigate it at excessive cost to themselves or others, or excusably ignorant of contributing. These limitations motivate a hybrid framework where PPP, which emphasizes holding those causing large-scale problems accountable, is balanced by the Ability to Pay Principle (APP), which emphasizes efficiently managing such problems. In this framework, improving wastewater treatment and distributing associated financial costs across water consumers or taxpayers stand out as promising responses to pharmaceutical pollution from human use. However, sound policy depends on empirical considerations requiring further study.

## Introduction

While pharmaceuticals are essential to human and animal well-being, their release into the environment is a source of growing concern. Emissions of pharmaceutical residues may occur during drug manufacturing, via urine and faeces following use in humans or domestic animals, in some cases via use on plants, and through inappropriate disposal of unused drugs (Boxall et al., 2012). The great majority of active pharmaceutical ingredients (APIs) are designed to interact already at low concentrations with target molecules in humans or pathogens, molecules that are often well conserved across a range of different non-target organisms (Gunnarsson et al., 2008). Accordingly, there is a growing body of evidence for effects on wildlife associated with exposure to various APIs, including reproductive disturbances, behavioural changes and disruption of organ development (Oaks et al., 2004; Kidd et al., 2007; Brodin et al., 2013; Näslund et al., 2017). Environmental emissions of antibiotics are particularly worrisome from a human health perspective because they can create a selection pressure favouring resistant bacterial strains (Bengtsson-Palme et al., 2018; Larsson and Flach, 2022), potentially exacerbating antimicrobial resistance, a major global threat to public health and economic development (WHO, 2015; World Bank, 2017). These concerns have recently led key international actors to emphasize the need for policy measures tackling pharmaceutical pollution (European Commission, 2019, 2022; OECD, 2019; European Parliament, 2020, 2021; WHO/FAO/OIE, 2020).

This paper contributes to the development of feasible and ethically justifiable action in this area, focusing on pollution from human use of pharmaceuticals via household and hospital wastewater.^1^ Specifically, we consider the *allocation of responsibility* between key actors for managing this problem. Many different management options exist (Boxall et al., 2012; Pruden et al., 2013; OECD, 2019). *Source-directed* measures mainly target pharmaceutical companies and concern the development of drugs that are environmentally ‘benign by design’. *Use-orientated* measures mainly target health professionals and patients and seek to reduce consumption indirectly limiting the subsequent environmental release of residues. *End-of-pipe* measures aim at removing pharmaceutical residues close to the point of entry into the environment, e.g., through waste collection or wastewater treatment. Specifically, while existing wastewater treatment plants (WWTPs) are not typically equipped to effectively capture and remove pharmaceutical residues, some European countries and regions have recently started adapting them for this purpose (Margot et al., 2013; Rizzo et al., 2019). These different types of measures will engage different actors, and impose economic costs, risks and other burdens on them and others in complex ways (Kirschke and Kosow, 2022). Justifying the choice and design of measures therefore requires considering the normative questions of how such costs, risks and burdens should be distributed, and on what grounds.

Previous research has considered the allocation of responsibilities for managing several other pressing environmental and public health challenges, including climate change (Caney, 2010; Knight, 2011; Moellendorf, 2012; Page, 2012), ecosystem conservation (Armstrong, 2018), lack of access to medicines in the developing world (Barry and Raworth, 2002; Malmqvist, 2016) and pollution from drug manufacturing (Nijsingh et al., 2019; Malmqvist and Munthe, 2020). By contrast, how responsibilities for managing pharmaceutical pollution from human use should be allocated remains an unexplored yet critical issue (Larsson et al., 2018).

The present paper seeks to fill this gap. Specifically, we examine the proposal—recently advanced by the European Federation of National Associations of Water Services (EurEau) (EurEau, 2019a,b, 2020; Deloitte, 2020) and endorsed by the European Parliament (2020) and Commission (2022)—that responsibilities for addressing pharmaceutical pollution from human use should be allocated using the Polluter Pays Principle (PPP). We challenge the suitability of PPP as sole rationale for allocating responsibility in this area, propose a more plausible rationale combining PPP with the Ability to Pay Principle (APP) and explore the practical implications of this shift in rationale.

The next section specifies the question under discussion as one concerning the distribution of ‘remedial responsibility’. The subsequent section analyses the role of PPP as the default framework for such distribution in recent European policy conversations concerning pharmaceutical pollution. Thereafter (in ‘Limitations of PPP’) we raise four challenges to PPP in this context. Meeting these challenges, we then argue (in ‘The way forward’), requires a hybrid framework where the backward-looking considerations of accountability and blame captured by PPP are tempered by the forward-looking considerations of efficiency captured by APP. Such a framework makes sound policy dependent on empirical considerations that require further study, which we conclude by briefly highlighting.

Four remarks on scope and method: First, while pharmaceutical pollution is a global concern, we focus on the European context. This is because, as mentioned, key European actors have recently proposed applying PPP to this problem, and because this principle is influential in European environmental regulation. While some of our findings may apply more broadly, the justifiability and feasibility of management options likely vary considerably in a global perspective.

Second, to focus specifically on responsibilities for managing pharmaceutical pollution from human use, we set aside other sources of pharmaceutical pollution, including animal use, pollution from manufacturing and inappropriate disposal of unused drugs. We also set aside pollution from medically inappropriate or excessive human use, e.g. self-medication with antibiotics and other prescription drugs (Grigoryan et al., 2019). While such use may contribute to an environmental impact, there are strong independent reasons to curb it (namely, patient safety and the need to preserve antimicrobial effectiveness). Managing pollution from appropriate human consumption may remain important in contexts where these other factors are already satisfactorily controlled (e.g. through effective antibiotic stewardship and pharmaceutical waste disposal). Thus, the allocation of responsibility for that management warrants consideration in its own right.

Third, our primary focus is the *rationale* (or justification) for allocating responsibility, i.e. the reasons or principles that make such allocation defensible. Such a rationale is necessary to identify responsible actors, but not sufficient. Empirical evidence is needed too, the nature of which depends on the chosen rationale. Thus, while some relevant evidence may presently be unavailable, the choice of rationale is important because it helps determine what evidence should be produced.

Fourth, we distinguish between two different ways in which the rationales under consideration (PPP and APP) can be used: (i) to justify the *choice of management option(s)* and (ii) to justify the distribution of *costs of management*. When used in the first way, they identify actors responsible for taking action to manage pharmaceutical pollution from human use. In effect, this amounts to choosing between source-directed, use-orientated and end-of-pipe measures since different measures engage different actors. When used in the second way, the rationales identify actors responsible for paying financially for the selected measure(s). While easily conflated, these uses should be carefully distinguished. It may well be that one actor (or group of actors) should do the management, whereas other actors should help bear the economic costs involved.

## Allocating Responsibility

The problem of pharmaceutical pollution from human use structurally resembles other pressing societal challenges, e.g. climate change. There is an undesirable situation (here, the presence in water bodies of pharmacologically active substances at potentially harmful levels) that needs to be addressed. However, it is unclear which among several actors (here, drug producers, health professionals, patients, WWTP operators, etc.) should address it. Thus, it needs to be determined who ought to shoulder this burden, and why.^2^

Philosophically, the problem concerns the distribution of *remedial responsibility* (Miller, 2001). Such responsibilities are defined by certain features: they are (a) *special*, i.e. not borne equally by all actors, (b) *normative*, i.e. they give rise to requirements to act and (c) *forward-looking*, i.e. geared towards producing an outcome, towards putting some ‘bad situation right’ (Miller, 2001: 454). Thus, to assign remedial responsibility for a ‘bad situation’ to an actor is to single out that actor from others as being required to engage in efforts to improve that situation.^3^

Since such efforts are costly or burdensome, some justification is needed for requiring actors to engage in them. Standard reasons used for this justification refer to moral responsibility, causal contribution, community and capacity (Miller, 2001; Björnsson and Brülde, 2016). That is, I may be required to improve some bad situation because its occurrence is my fault (moral responsibility), because I innocently played a role in causing it (causal contribution), because I have some special pre-existing responsibility or relationship to those affected by it (community) or because I am especially capable of improving it (capacity).^4^

These general grounds of responsibility find specific expression in environmental law and policy. PPP states that the cost of addressing damages caused by pollution should be distributed to actors in proportion to how much they pollute, thus appealing to *moral responsibility* and/or *causal contribution*, whereas APP states that such costs should be distributed in proportion to actors’ ability to pay, linking to considerations of *capacity* (Caney, 2010; Moellendorf, 2012).

The next section outlines PPP’s central role in recent European conversations about the allocation of responsibilities to address pharmaceutical pollution from human use. Thereafter we first highlight important limitations of PPP when applied to this case and then discuss how these limitations can be overcome by a hybrid rationale combining PPP and APP.

## PPP and Pharmaceutical Pollution from Human Use

PPP is invoked as rationale for allocating responsibilities for waste and wastewater management in several binding policy documents within the EU. For instance, the Water Framework Directive states that ‘environmental damage should, as a priority, be rectified at source and that the polluter should pay’ (2000/60/EC, preamble §11), whereas Directive 2018/851 amending the Waste Framework Directive (2008/98/EC) states that ‘[i]n accordance with the polluter-pays principle, the costs of waste management, including for the necessary infrastructure and its operation, shall be borne by the original waste producer or by the current or previous waste holders’ (Article 14). Together, these statements locate responsibility (for paying the costs and organizing necessary actions and facilities) for waste management as closely as possible to the source of the waste producing chain. In addition, both documents refer to the *Extended Producer Responsibility Principle* (EPR): ‘producers of products bear financial or financial and organizational responsibility for the management of the waste stage of a product’s life cycle including separate collection, sorting and treatment operations’ (Ibid, preamble §14). EPR legally operationalizes PPP in a way that allows control of the whole life cycle of a product by pressure on *producers*, via the basic accountability of *polluters* implied by PPP. In this way, PPP provides a framework of legal accountability through the imposition of financial and organizational responsibilities to prevent and mitigate environmental damage across the EU.

Recently, PPP and EPR have figured prominently in European policymaking related specifically to pharmaceutical pollution. In 2019, the European Commission (2019) set out to explore the potential role of the EPR framework for addressing this problem. The 2020 European Parliament resolution on a strategic approach to pharmaceuticals in the environment stresses PPP and EPR as instruments for addressing pollution from both manufacturing and use of pharmaceuticals (European Parliament 2020: §5), a stance repeated in the 2021 resolution on a pharmaceutical strategy for Europe (European Parliament, 2021: §157). And in October 2022, the Commission (2022) proposed revising the Urban Wastewater Treatment Directive (91/271/EEC) to, *inter alia*, require member states to ensure that pharmaceutical and cosmetic residues are removed at urban WWTPs (Article 8) and that costs for necessary WWTP upgrades are borne by pharmaceutical and cosmetic producers under EPR (Article 9).

The most developed case for applying PPP to pharmaceutical pollution from human use has been presented by EurEau, which represents European national WWTP operators. EurEau draws extensively on PPP to argue that the primary responsibility to manage this problem and bear associated costs falls on polluting actors—pharmaceutical producers, regulators, healthcare providers and patients—not on WWTP operators (EurEau, 2019a,b, 2020; Deloitte, 2020). In effect, EurEau uses PPP to advocate priority to management options that engage these actors, i.e. source-directed and use-orientated measures, citing a recent OECD (2019) report that makes similar recommendations. This stance has later been echoed by some European national water service providers (e.g. Svenskt Vatten, 2021). While EurEau acknowledges that end-of-pipe solutions in the form of improved treatment at WWTPs may have some role to play (EurEau, 2019b), these are characterized as ‘a means of last resort’ (EurEau, 2019a: 3), whereas ‘control-at-source measures should have absolute priority’ (EurEau, 2020: 8).

In addition to citing PPP to establish that end-of-pipe solutions are of secondary importance, EurEau (2019b, 2020) draws on PPP to argue that, insofar as such solutions are necessary, the cost for them should mainly be borne by the pharmaceutical industry. Upgrading WWTPs to remove pharmaceutical residues will generate financial costs, the magnitude of which is contested (Naturvårdsverket, 2017; Svenskt Vatten, 2021) and varies between techniques used (Rizzo et al., 2019). EurEau advocates applying EPR (hence by extension PPP) to allocate these costs to ‘those producing or making available on the market products that emit micropollutants and microplastics’ (EurEau, 2020: 6) rather than to water operators or water consumers.

EurEau thus uses PPP in both ways distinguished in the introduction: to support the choice of ‘upstream’ over ‘downstream’ management options, and to justify allocating the cost for necessary downstream solutions (WWTP upgrades) mainly to pharmaceutical producers. They characterize this application of PPP both as *fair*, because it assigns responsibility where it is due (to the polluters), and as *effective*, because it encourages upstream actors to avoid or reduce polluting behaviour (e.g. by developing ‘green’ pharmaceuticals and adopting sustainable drug prescription, consumption and disposal practices) (Deloitte, 2020; EurEau, 2020). This echoes two widely acknowledged general advantages of PPP and the underlying moral responsibility principle (Miller, 2001; Moellendorf, 2012; Armstrong, 2018). First, PPP is sensitive to how harms that need to be addressed come about, reflecting a ‘you broke it, you fix it’ logic that is intuitively appealing and familiar from various moral and legal practices. Second, by holding those causing harm responsible, it potentially disincentivizes harmful or reckless behaviour. While these are genuine advantages, PPP also has important disadvantages which, we argue, motivate giving it a more limited role than its proponents in the present discussion suggest.

## Limitations of PPP

We will highlight four significant ethical and practical challenges to the application of PPP to pharmaceutical pollution from human use: the indeterminacy problem, costs of avoiding pollution, the inability of polluting actors and excusable ignorance. Taken together, these challenges make PPP unsuitable as sole rationale for allocating responsibility in this area, calling for a reconsideration of European water operators’ PPP-based case for prioritizing upstream action and allocating costs of downstream action to putative polluters.

### The Indeterminacy Problem

The indeterminacy problem arises due to difficulties in establishing the causal chain of events producing harms that need to be addressed (Miller, 2001; Caney, 2010). According to PPP, responsibility is borne by polluters, in proportion to how much they pollute. To apply PPP, we therefore must trace different actors’ contributions to the pollution and assess their relative magnitude or importance. In the case of pharmaceutical pollution from human use, this means accounting for the behaviour of a multitude of actors, including, among others, manufacturers, distributors, authorization agencies, healthcare providers, physicians and patients. This is empirically challenging because many of the contributions are likely difficult to measure. However, the problem goes deeper: even with accurate and complete empirical information, it needs to be determined which contributions should count in the first place and how to individuate and compare them. A difficulty here is that different contributions to pharmaceutical pollution from human use are highly interdependent. A patient’s excretion of drug residues presupposes that a manufacturer produces the drug, that an authorization agency approves it, and (often) that a healthcare system procures/subsidizes it and that a physician prescribes it. Conversely, these other actors do not contribute to pollution (via excretion) unless the patient ingests the drug. This makes it difficult to disentangle different actors’ contributions in the way necessary for applying PPP, leaving it indeterminate where responsibility should lie.

This indeterminacy is compounded by the fact that omissions to act are sometimes considered causes for purposes of attributing moral and legal responsibility (Miller, 2001). Specifically, the EU understanding of PPP considers both ‘active’ polluting practices and failures to take action to mitigate pollution as falling under the principle (Hinteregger, 2008). In effect, not only manufacturers, authorization agencies, healthcare providers, physicians and patients may count as polluters. A WWTP operator that fails to take steps to improve the removal of pharmaceutical residues from wastewater may *also* qualify as polluter, but by omission,^5^ and thus, under PPP, be required to take such steps or help fund them.

Admittedly, omissions are not always considered causes of relevance for attributing (moral or legal) responsibility, but mostly count as such when they represent a failure to fulfil some prior obligation, i.e. in cases of culpable negligence (Miller, 2001). However, WWTP operators are arguably under such an obligation, since their socially sanctioned task is to help secure clean waterways. A failure to contribute to this task by taking steps to mitigate pharmaceutical pollution may thus be construed as a relevant cause of the pollution.

The indeterminacy problem challenges PPP in two related ways. First, if we cannot identify some actors as more salient polluters than others, we cannot proportionately assign responsibility. This makes PPP hard to apply, limiting its capacity to guide action. Second, and more fundamentally, distributing responsibility based on actors’ contributions to a problem seems *arbitrary* absent a clear picture of the relative magnitude or importance of these contributions. Actors who are assigned larger responsibilities may object, not unreasonably, that they are required to carry greater burdens than others without proper justification.

In response, some might dismiss indeterminacy as a mere theoretical worry because, in practice, EPR specifies PPP by unambiguously assigning responsibility to producers. This response meets the first of the challenges because EPR does single out some polluters as particularly salient. However, it does not meet the second, more fundamental challenge. We still need to ask why pharmaceutical producers rather than other putative polluters *should* be singled out given the complexity of interdependent contributions highlighted above, i.e. what justifies specifying PPP in this way. Here, appealing to EPR clearly begs the question.

The indeterminacy problem challenges PPP both as justification for the choice of management options and as rationale for allocating associated costs. If the most salient polluters cannot be singled out it cannot be determined what actors are mainly responsible for implementing mitigation measures, nor how large costs each actor should bear. Proportionally allocating costs for WWTP upgrades is particularly difficult given that such upgrades would simultaneously remove many pollutants, which reach WWTPs via partly distinct causal pathways involving distinct actors. Performing accurate, quantitative comparisons of the environmental impact associated with each pollutant is exceptionally challenging.

### Costs of Avoiding Pollution

This challenge links to a common (moral and legal) notion that there are limits to what actors may reasonably be expected to do to avoid causing harm or mitigate harm they cause (Caney, 2010). This concerns both costs or burdens to the actor herself and indirect costs of her action to others. The options that contributors to pharmaceutical pollution from human use have for avoiding or reducing their contribution normally involve costs of either or both kinds. Just like other APIs, those associated with environmental risk are also important for clinical practice and public health. This means that the indirect costs would be high if drug manufacturers were to avoid pollution from use by reducing or even ending production of such APIs. This is not usually considered an option precisely because of the steep potential burden on patients: lack of access to needed medicines. Perhaps more realistically, drug companies could invest in developing new APIs that are ‘benign by design’, i.e. pose less environmental health risks. However, while this could help phase out potentially polluting drugs in the long term, it will likely play at most a minor role in the nearest decades given the time frame and scientific challenges of bringing new APIs to market.^6^ In the short-to-medium term, it is sometimes possible to substitute an active ingredient associated with high environmental risk with a clinically equivalent alternative posing lower risk. For example, diclofenac, which can affect the kidneys in vultures and fish (Oaks et al., 2004; Näslund et al., 2017), may sometimes be replaced with another similarly acting anti-inflammatory drug that is less toxic to fish (Näslund et al., 2020). Substitution could also involve different administration forms where e.g. gels for topical applications could lead to higher environmental releases than pills containing the same active substance (Bielfeldt et al., 2022). However, for the great majority of situations, changing the active ingredient or the administration form comes with a differential risk of side effects and/or differential opportunities to achieve the desired effect in the patient. In these cases, the options for avoiding or reducing pollution (bracketing improper prescription, use and disposal of drugs; see ‘Introduction’) are to rely on a clinically inferior alternative or abstain from using the polluting drug—both of which are clearly burdensome to patients.

Of course, substantial cost is not necessarily a reason against taking action to avoid or mitigate harm, since alternative mitigation approaches may be equally or more costly. The point is that since PPP assigns responsibility exclusively based on contributions to pollution, it is insensitive to such costs. PPP does not consider the comparative costliness of options available to different polluters when selecting among these options, nor the possibility that options available to non-polluters may involve lower cost than any of these. PPP does not, for instance, allow us to compare the potential health burdens of different actions targeting drug development, prescription and use to each other, nor to the monetary costs of upgrading WWTPs to address pharmaceutical pollution end-of-pipe, when determining how to address this problem. However, such comparisons seem vital for selecting justifiable action in this area.

The cost challenge affects the use of PPP to select management options, since the options currently available to upstream polluting actors would potentially impose substantial health burdens on patients. Potentially the challenge also concerns the use of PPP to justify allocating economic costs to these actors, but it seems less pressing here. Some such actors (e.g. healthcare systems or financially insecure patients) may face powerful competing demands on their resources making them unable to bear costs proportional to how much they pollute without foregoing other important expenses. However, other potentially polluting actors (e.g. financially secure patients) may be so numerous and contribute so little individually to the pollution that each can bear proportional costs without incurring significant burdens.

### Inability of Polluters

The cost challenge leads naturally over to another challenge to PPP. Many authors have noted that PPP (or the underlying causal contribution or moral responsibility principles) is susceptible to allocating responsibility to actors that, though they have (culpably) polluted or otherwise caused harm, are unable to address that harm (Miller, 2001; Caney, 2010; Young, 2011). In consequence, the harm will remain unaddressed, compromising the purpose of assigning responsibility in the first place (Miller, 2001). Of course, mere *reluctance* among polluters to engage in pollution control is no justification for failing to do so. However, in some cases the option to avoid or reduce pollution may simply not exist, e.g. when a pharmaceutical producer cannot switch from a conventional drug to a ‘green’ alternative because no such alternative has yet been developed. In other cases, the option may exist but be effectively blocked by other actors. For instance, the costlier reducing the use of polluting drugs is for patients (see ‘Costs of Avoiding Pollution’) the likelier are they and their treating physicians to resist such reductions, potentially making it infeasible for healthcare decision-makers to implement them even if they are formally decided. Thus, in a context where drugs are largely properly prescribed, used and disposed of, there are likely limits to how far healthcare institutions can go towards reducing use-related pharmaceutical pollution.

Of course, the observation that some of polluters’ options are infeasible does not show that all of them are. Rather, the point is this: the feasibility of such options lacks relevance for PPP due to its exclusive concern with contributions to pollution, which limits its ability to allocate responsibility in a way that ensures the pollution is effectively addressed.

The inability challenge mainly affects PPP as a justification for the choice of management options, as key polluters may lack realistic ways of implementing the necessary changes. However, as with the cost challenge, it seems less as damaging to PPP as rationale for allocating economic costs. Some polluters (e.g. healthcare institutions and financially insecure patients) may face financial constraints that make it not only burdensome but infeasible to proportionally pay for pollution control, whereas this is less plausible for polluters who are better off and whose individual contributions are small.

### Excusable Ignorance

The excusable ignorance objection to PPP maintains that it is unfair to allocate the burdens of pollution control to polluters if they are unaware, and could not reasonably be expected to be aware, of causing the pollution. More fundamentally, causal contribution to a problem is in most cases considered insufficient grounds for responsibility to address that problem; knowledge or culpable ignorance of the contribution is needed too (Miller, 2001).

Now many contributors to pharmaceutical pollution from human use are or should be aware of their contribution, and so cannot plausibly appeal to excusable ignorance to refuse bearing the burdens of pollution control. Certainly, it should be evident to drug producers that a variety of the APIs they produce risk causing harm when released into waterways. Similarly, regulators and healthcare providers know or can reasonably be expected to know about such risks and their role in creating them. WWTP operators, too, clearly seem aware of these risks, given the recent attention that organizations representing them have devoted to this issue (EurEau, 2019a,b, 2020; Deloitte, 2020; Svenskt Vatten, 2021). The same arguably goes for individual prescribing physicians, but this is less obvious as they may not have been educated about such risks and may have limited opportunities to learn about them in their professional roles.

While the excusable ignorance objection cannot plausibly be raised on behalf of these actors (possibly excluding physicians), it is forceful when raised on behalf of patients. This is because patients cannot reasonably be expected to be aware of risks associated with use-related pharmaceutical pollution as they generally and quite appropriately rely on public agencies’ and healthcare providers’ expertise concerning the drugs they consume. Given their excusable ignorance, it seems unfair to apply PPP to allocate the burdens of pollution control to them. Such allocation would be *direct* if patients were expected to bear management responsibility by choosing less over more polluting alternatives when purchasing medicines,^7^ or assigned economic costs by having to pay more for the latter. More importantly, however, measures taken by *other* putative polluters will typically *indirectly* impose burdens on patients. Avoiding or reducing the production, approval or prescription of medically indicated but potentially polluting drugs will—as highlighted above (see ‘Costs of Avoiding Pollution’)—potentially negatively affect patients’ health.^8^ The excusable ignorance objection challenges both the direct and indirect imposition of burdens on patients under PPP.

There are two ways of sidestepping this objection, defending the imposition of burdens on patients despite their ignorance. The first is to interpret PPP as based on causal contribution alone, rather than causal contribution combined with knowledge or culpable ignorance. PPP would then be understood as a *strict liability* principle: ‘if people engage in activities which jeopardize other people’s fundamental interests [then] they should bear the costs of their actions even if they were excusably ignorant of the effects of their actions’ (Caney, 2010: 208; cf. Shue, 1999). However, while strict liability has legal precedent in certain specific contexts, it is often considered unfair as a general moral principle because it holds people responsible for outcomes they cannot predict or avoid (Caney, 2010; Moellendorf, 2012). The second approach would be to inform patients about use-related pharmaceutical pollution and their role in it. Effective and accessible information campaigns could ensure that patients are either aware of this problem or no longer *excusably* ignorant since the relevant information is easily and widely available. This might be feasible in the case of *directly* allocating management responsibility or economic costs to patients purchasing medicines since the information could be included in product leaflets or supplied by pharmacists. However, it seems much harder to ensure that patients *indirectly* affected by measures taken by other actors to reduce the production, approval or prescription of polluting drugs are adequately informed. Thus, the excusable ignorance objection remains a challenge to PPP as rationale for the choice of such management options even granted the possibility of informational efforts.

### Summing-up

We have highlighted four challenges to PPP as sole rationale for distributing responsibility for managing pharmaceutical pollution from human use. First, difficulties in determining who the relevant polluters are in this case make PPP hard to apply non-arbitrarily. Moreover, PPP is susceptible to allocating responsibility to actors who are unable to address the harm they cause, who can only address it at excessive cost to themselves or others, or who are excusably ignorant of causing it. All four challenges apply to the use of PPP as guide to selecting management options, whereas the use of PPP as justification for allocating economic costs is mainly vulnerable to the first one. This suggests that European water operators’ appeal to PPP requires significant modification, especially as justification for prioritizing source-directed and use-orientated measures but also as rationale for allocating costs of end-of-pipe action to upstream actors.

More fundamentally, these challenges arise because PPP assigns responsibility exclusively on *retrospective* grounds, by asking who caused or is causing a problem. This provides reason to include *prospective* considerations when assigning responsibility in this area, asking who is best placed to solve or mitigate the problem. This is our task in the next section.

## The Way Forward

Given the limitations of a purely retrospective approach, it is worth starting by considering a purely prospective alternative, which, in the context of environmental law and policy, is represented by APP. As noted above (see ‘Allocating Responsibility’), APP is the expression of a more fundamental capacity principle, according to which the responsibility to address a problem should be allocated to the actor(s) who are able to address it (most) efficiently (Miller, 2001; Gosseries, 2004). Once APP is understood in this way, it becomes clear that its name may be somewhat misleading. An actor’s capacity to address a problem has two dimensions: *effectiveness* (the likelihood of having an impact, and the magnitude of that impact) and *cost* (the burden the actor incurs or imposes on others by addressing the problem) (Miller, 2001). Thus, the ‘pay’ in APP should be understood as including not only (direct and indirect) financial costs, but also other burdens to the actor and other parties, whereas ‘ability’ includes both the ability to shoulder these burdens and the ability to accomplish effective management.

APP is attractive in that it prioritizes ensuring that ‘bad situations’ are actually improved, independently of how they come about, reflecting the overarching purpose of allocating responsibility. This represents an advantage over PPP, which, as argued in the preceding section, lacks precisely this feature. Also, APP has a certain egalitarian appeal as it tends to assign greater burdens to the better off than to the worse off (Armstrong, 2018). On the other hand, APP lacks the features that make PPP attractive. Since it is insensitive to how harms that need to be addressed happen, it finds no support in the intuitively plausible ‘you broke it, you fix it’ logic underpinning PPP and does not disincentivize harmful or reckless behaviour.

Since both PPP and APP have notable strength and weakness, and since their respective strengths and weaknesses mirror each other, it is plausible to consider them as complementary rather than rival principles (cf. Miller, 2001). Consequently, hybrid or pluralist views—which are concerned *both* with how problems that need to be addressed come about (reflecting PPP) and with how to address them most efficiently (reflecting APP), and perhaps with further considerations—are widely endorsed by theorists and policymakers, in the climate change context (e.g. United Nations, 1992; Caney, 2010; Knight, 2011) and elsewhere (Armstrong, 2018). Similarly, we contend that a hybrid approach combining PPP and APP provides a better rationale for distributing responsibilities than PPP alone in the case of pharmaceutical pollution from human use too.

While APP and PPP can be combined in different ways, we will briefly sketch a framework specifically designed to overcome the limitations highlighted above. In this framework, APP is used to (i) *specify*, (ii) *constrain*, (iii) *complement* and (iv) *override* the recommendations provided by PPP. Consider each function in turn:

(i) While contributions to pollution remain relevant in a hybrid framework, we have seen that this consideration alone yields indeterminate guidance because different contributions cannot be readily disentangled and because their relative magnitude and importance are hard to assess. A hybrid framework overcomes this indeterminacy problem by using PPP to demarcate the broader class of candidates for responsibility assignment (all polluters, including polluters by omission; see ‘The Indeterminacy Problem’), and then applying APP to discriminate within that class. That is, APP is used to single out, from among the polluters, those who can contribute to reducing or mitigating the pollution most efficiently. There is thus no need to determine just how much each polluter contributes (as would be necessary under PPP alone).
(ii) Singling out capable polluting actors also addresses the cost challenge, by taking the burdens on the actor and others into account when assigning responsibility, and the inability challenge, by considering whether actors are capable of having a meaningful impact. Here APP constrains PPP, ruling out responsibility assignments that impose excessive burdens (cf. Caney, 2010; Armstrong, 2018) or are practically ineffective.
(iii) If capable polluting actors cannot be clearly identified, a hybrid framework licenses assigning responsibility to other actors based on capacity alone. Here PPP lacks traction and APP is used as an alternative rationale in its own right, addressing the inability problem (cf. Caney, 2010; Armstrong, 2018).
(iv) If capable polluters can be identified, but their options for addressing the pollution are considerably less feasible or more (directly or indirectly) burdensome than options available to other actors, a hybrid approach again licenses assigning responsibility to these latter actors based on capacity alone. Here PPP remains applicable but is outweighed by the concern for efficiency captured by APP, again addressing the cost and inability challenges.

In this way, attractive features of PPP are preserved, while the indeterminacy, cost and inability challenges to PPP are addressed. The excusable ignorance objection is not fully defused, though, since excusably ignorant polluters could be assigned burdens also under a hybrid framework. However, the problem is significantly less pressing in this framework, partly because the constraining function helps limiting these burdens, and partly because the complementing and overriding functions ensure that the main rationale for assigning them will often not be contribution but capacity—a rationale that is not susceptible to this objection (Moellendorf, 2012).

Consider how this approach applies to the case at hand. As regards the choice of management options, we have seen that the most salient upstream approaches either have limited feasibility (e.g. developing ‘green’ pharmaceuticals), or potentially impose considerable health costs on patients (e.g. reducing medically indicated drug prescription and use when no clinically equivalent alternatives exist). On a hybrid view, these are weighty drawbacks. Moreover, on this view, who contribute most to a problem is not decisive for determining who ought to manage it. Taken together, these considerations make the option of upgrading WWTPs to better remove pharmaceutical residues downstream a strong candidate. Such upgrades, which are already being implemented in parts of Europe (Margot et al., 2013; Rizzo et al., 2019), would avoid the potential health burdens associated with upstream approaches. Also supporting this option is the potential ‘collateral benefit’ of improved removal of various non-pharmaceutical pollutants (Larsson and Flach, 2022). While a conclusive case for prioritizing WWTP upgrades must rely on empirical evidence (more on this below), the hybrid approach sketched here lends it tentative support.

This prompts the question who should bear the financial costs of such upgrades. The answer here is less straightforward. On the one hand, like PPP, a hybrid framework gives weight to whether actors contribute to pollution, consistent with the proposal that drug producers pay (EurEau, 2019b, 2020; Svenskt Vatten, 2021; European Commission, 2022). Assigning costs to producers has two potential advantages in this framework. First, it could incentivize investment in developing ‘green’ APIs. Second, the intuitive appeal and legal precedents of having producers pay for pollution control may help ensure social legitimacy and political feasibility.

On the other hand, unlike PPP, a hybrid framework only assigns the cost of pollution control to polluters if they can absorb it without excessive setback to their own or other actors’ interests. It is unclear that having drug producers pay meets this condition. The crux is not primarily the *direct* costs to producers, but the *indirect* burdens on health systems and patients that these costs may generate. Drug producers are for-profit enterprises operating in a market setting, so having them pay for WWTP upgrades will incentivize them to recoup their increased costs through higher drug prices or to leave markets where such requests are made (Nijsingh et al., 2019). This could potentially limit patients’ access to medicines. Even if a drug is publicly subsidized, a higher price may affect access to *that* drug by reducing society’s willingness to pay for it or access to *other* drugs since subsidizing it would claim a larger share of society’s pharmaceutical budget. Societies could of course decide to refuse price increases driven by producers’ costs for WWTP upgrades, but this would again risk access by discouraging producers from selling polluting drugs on national or regional markets. Given the clinical importance of these drugs, diminished access to them may have real health impact. Diminished access may also result, though more directly, if patients or health systems are assigned the cost of WWTP upgrades through fees or taxes on polluting drugs.

Though not conclusively ruling out having drug producers, patients or health systems (partially) bear the cost of WWTP upgrades, these considerations support looking beyond putative polluters when allocating these costs. One option is to offer water operators state subsidies funded through general taxation or other economic incentives for undertaking such upgrades,^9^ another is to have water consumers pay through increased water fees. Both options avoid the potential health consequences of targeting putative polluters and limit the burden on each individual by widely dispersing the costs.

This discussion shows how shifting the rationale for allocating responsibility also shifts what options for addressing pharmaceutical pollution from human use warrant consideration. In a hybrid framework, upgrading WWTPs to manage the problem end-of-pipe and distributing associated costs across taxpayers or water consumers stand out as promising approaches. These suggestions remain preliminary, though, and further analysis requires attention to empirical matters. These include the effectiveness of different techniques for removing pharmaceutical residues at WWTPs (Margot et al., 2013; Rizzo et al., 2019), economic costs of implementing these in different settings (Naturvårdsverket, 2017; Rizzo et al., 2019; Svenskt Vatten, 2021), appropriate ways of incentivizing water operators to take action (cf. Nijsing et al., 2019; Malmqvist and Munthe, 2020), the ability of different actors to shoulder costs of WWTP upgrades without sacrificing other important interests, and the feasibility and social legitimacy of allocating these costs to them. Moreover, potential side effects of WWTP upgrades should be considered, including the ‘collateral benefit’ of improved removal of non-pharmaceutical pollutants (Larsson and Flach, 2022) and potentially increased energy use and CO_2_ emissions (Rizzo et al., 2019). While assessing these issues is beyond the scope of this paper, we have hopefully provided a plausible rationale for analysing them further and for identifying further areas of empirical inquiry.

## Conclusions

Policymakers must consider how responsibilities for managing risks created by pharmaceutical pollution from human use should be distributed between actors. We have assessed whether PPP is a suitable basis for such distribution, as recently suggested by European water operators and the European Parliament and Commission. While recognizing that PPP has notable advantages, we have highlighted four challenges with applying it to this case. First, since it is unclear who the most salient polluters are, PPP fails to unambiguously assign responsibility. Second, insofar as PPP does identify responsible actors, these may be unable to avoid or mitigate their contribution or, third, only able to do so at excessive cost to themselves or others. Fourth, PPP is susceptible to assigning burdens to actors who cannot fairly be held responsible due to excusable ignorance. These limitations motivate a hybrid framework where PPP, which emphasizes holding those causing large-scale problems to account, is balanced by APP, which emphasizes the efficient management of such problems. While sound policy in this area depends on empirical issues calling for further study, this framework tentatively supports managing pharmaceutical pollution from human use by improving wastewater treatment. The allocation of associated costs remains to some extent an open question, but the importance of preserving access to clinically important medicines supports distributing these across water consumers or taxpayers.

Two final caveats: First, the challenges we have raised for PPP concern its role as rationale for policy on pharmaceutical pollution from human use, not its suitability in other areas, e.g. climate change or pollution from drug manufacturing. Second, some readers may disagree with (aspects of) the hybrid framework we have sketched or some practical suggestions we have made. However, even these readers should recognize the insufficiency of PPP as guide for policy in this area and the need to supplement or amend it, albeit perhaps in other ways.

## Footnotes

1 Globally, other pathways for pharmaceutical pollution from human use are common, e.g. open defecation and pit latrines. However, given our European focus (see below) we set these aside, focusing on the wastewater pathway that predominates in this context.

2 Not all undesirable situations require improvement efforts necessitating the allocation of associated burdens. However, consistent with recent policy discussions in this area, we assume that pharmaceutical pollution from human use does require such efforts.

3 Unless otherwise stated, we intend the terms ‘responsibility’ and ‘responsible’ to refer to this kind of responsibility.

4 The *structure* of remedial responsibilities should be distinguished from their *grounds*. While their structure is prospective (they aim at producing an outcome), the grounds or rationale for assigning them to actors may be retrospective (such as what the actors have done). The concerns about PPP elaborated below arise because PPP assigns such responsibilities on purely retrospective grounds.

5 Indeed, any action may be truthfully described as an omission of other, alternative actions, and any omission can be truthfully described in terms of an action that could have been performed instead (Woollard and Snyder, 2021).

6 Innovative drugs approved in the USA between 2010 and 2020 had spent 5–20 (median 8.3) years in clinical development (Brown et al., 2022), which, in the case of new APIs, is preceded by several years of discovery and preclinical research. As an extreme example of slow and challenging drug development, no new antibiotic class targeting Gram-negative bacteria has been approved in several decades, despite great efforts (Kingwell, 2018).

7 Such choices do not impose health burdens if the options are clinically equivalent. However, clinically equivalent alternatives do not exist for many polluting drugs (see ‘Costs of Avoiding Pollution’).

8 Again, we assume that such measures are implemented in a context where drugs are appropriately prescribed, consumed and disposed of (see ‘Introduction’).

9 Compare discussions about incentivizing pharmaceutical companies to address pollution from drug manufacturing (Nijsingh et al., 2019; Malmqvist and Munthe, 2020).
